# Evaluation of PD-1 T lymphocytes in bronchoalveolar lavage in lung cancer compared to benign lung diseases

**DOI:** 10.1007/s10238-023-01146-6

**Published:** 2023-08-03

**Authors:** B. Hammer, C. Bal, M. Gysan, S. Zehetmayer, S. Geleff, A. Zech, M. Kramer, S. Ayazseven, M. Idzko, B. Mosleh, M. A. Hoda, D. Gompelmann

**Affiliations:** 1 Division of Pulmonology, Department of Internal Medicine II, Medical University Vienna, Währinger Gürtel 18-20, 1090 Vienna, Austria; 2 DZL Laboratory for Experimental Microbiome Research, Airway Research Center North (ARCN), German Center for Lung Research (DZL), Research Center Borstel, Borstel, Germany; 3 Center for Medical Data Science, Medical University of Vienna, Vienna, Austria; 4 Department of Pathology, Medical University Vienna, Vienna, Austria; 5 Department of Thoracic Surgery, Medical University Vienna, Vienna, Austria

**Keywords:** Lung cancer, Bronchoalveolar lavage, PD-1/PD-L1 pathway

## Abstract

The expression of the programmed cell death protein 1 (PD-1) has been shown to be markedly increased in tumor-infiltrating lymphocytes. However, the proportion of PD-1 + T cells in the bronchoalveolar lavage (BAL) of lung cancer patients has not been sufficiently evaluated so far. In this prospective study, the proportion of PD-1 + CD4 + as well as PD-1 + CD8 + T cells in BAL samples, isolated from patients with lung cancer, asthma or interstitial lung disease (ILD), were determined via flow cytometry and compared for differences. Bronchoalveolar lavage was performed in 34 patients (14 patients with lung cancer, 10 patients with asthma, 10 patients with ILD). The highest median proportion of PD-1 + CD4 + or PD-1 + CD8 + T cells were found in patients with ILD (83.1% [IQR 72.1; 87.5] and 73.8% [IQR 60.3; 86.3]) followed by patients with lung cancer (66.4% [IQR 59; 69] and 77.1% [IQR 35.8; 82.3]) and patients with asthma (61.3% [IQR 57.4; 70.5] and 57.3% [IQR 46; 65]). Thereby, the difference in the proportion of PD-1 + CD3 + CD4 + BAL cells between ILD patients and asthmatics was significantly different (*p* = 0.04). The proportion of PD-1 + CD4 + and PD-1 + CD8 + T cells in the BAL of patients with lung cancer did not differ significantly to patients with benign lung diseases. The highest proportion was observed in ILD patients suggesting further research to evaluate the role of the PD-1/PD-L1 pathway in ILD patients.

## Introduction

The programmed cell death protein 1 (PD-1) is an immune checkpoint molecule belonging to the immunoglobulin super family and is expressed on the surface of activated T cells, natural killer (NK) cells, B lymphocytes and monocytes. PD-1 can interact with its ligands programmed cell death 1 ligand 1 (PD-L1) and programmed cell death 1 ligand 2 (PD-L2) and thus inducing a suppression of immune responses. This interaction plays a critical role in cancer as tumor cells are capable of escaping immune surveillance by expression of PD-L1. It is known that PD-1 expression is markedly increased in tumor-infiltrating immune cells, which may promote tumor development [1, 2].

In recent years, immune checkpoint inhibitors (ICI)—antibodies that target PD-1 or PD-L1—have emerged as treatment modality in various types of cancer. ICIs block the interaction of PD-L1 on tumor cells with PD-1 on T lymphocytes thus preventing immunity suppression and subsequent tumor cell evasion. ICI therapy has been shown to improve the survival of patients with advanced metastatic lung cancer [3]. Several trials demonstrated a superior response to ICI particularly in patients with PD-L1 expression > 50% on tumor cells [4–6]. However, the correlation of high PD-L1 expression on tumor cells with treatment response is discussed controversially and may vary among different ICI agents [7].

Although increased numbers of T cells have been observed in bronchoalveolar lavages (BAL) of lung cancer patients [8, 9], there is limited data on the proportion of PD-1 + T lymphocytes in such BAL samples. Kwiecien et al. demonstrated an increased proportion of PD-1 + CD4 + and CD8 + lymphocytes in the BAL of lung cancer patients compared to the respective blood samples [10]. Moreover, patients with squamous cell cancer showed a higher proportion of PD-1 + BAL T cells compared to patients with adenocarcinoma. Kumagai et al. hypothesized that the frequency of PD-1 + CD8 + lymphocytes in relation to the frequency of PD-1 + regulatory T lymphocytes in the tumor microenvironment can predict the clinical efficacy of immunotherapies and is superior to PD-L1 tumor cell expression as indicator of treatment response [11].

While there is limited data on PD-1 + T cells in lung cancer, there is even less knowledge regarding PD-1 expression on T cells in patients with benign lung diseases. d'Alessandro and colleagues reported a high expression of PD-1 on T cells in BAL of patients with sarcoidosis [12]. Kronborg-White et al. described an expression of PD-L1 in epithelial cells in a subgroup of patients with IPF [13]. However, the proportion of PD1 + lymphocytes in patients with lung carcinoma compared to patients with benign lung diseases has not been fully elucidated yet.

As PD-1 expression on lymphocytes seems to be increased in tumor microenvironment, it can be hypothesized that the proportion of PD-1 + T cells is greater in BAL of lung cancer patients. The aim of this prospective study was to evaluate whether there is a difference in the proportion of PD1 + T cells in lung cancer patients compared to patients with benign lung diseases.

## Methods

Patients with lung cancer, asthma (obstructive ventilator disorder) or interstitial lung diseases (ILD; restrictive ventilator disorder) with indication for bronchoscopy were included in this prospective trial. All bronchoscopies with BAL, sample processing and flow cytometry were performed at the Medical University of Vienna (Austria). The ethics committee of the Medical University of Vienna approved the protocol of this study (1652/2020) and all patients gave written informed consent prior to study enrolment.

### Study subjects

In this prospective study, patients aged > 18 years with a pulmonary lesion suspicious for lung cancer, patients with asthma and ILD were enrolled. Patients with endobronchial tumor growth, concomitant pneumonia or acute exacerbation of asthma or ILD were excluded from this trial.

### Bronchoscopy, BAL sample processing and flow cytometry

All patients underwent bronchoscopy in sedation or general anaesthesia. BAL was performed by instillation of 200 ml saline in the middle lobe. BAL samples were kept on ice, filtered through gauze (Gazin, Lohmann & Rauscher, Austria) and centrifuged at 400 × g for 15 min at 4 °C. Erythrocyte lysis was performed when appropriate.

For flow cytometry, BAL cells were treated with Fc Block (clone Fc1.3216, BD Pharmingen, CA, USA) prior surface marker staining and DAPI (Invitrogen, Thermo Fisher scientific, CA, USA) for dead cell exclusion. Antibodies used were anti-human CD45-BV510 (clone HI30, BD Horizon, CA, USA), CD3-FITC (clone UCHT1, BD Pharmingen, CA, USA), CD4-PE-Cy7 (clone SK3, BD Pharmingen, CA, USA), CD8-ECD (clone SFCI21Thy2D3, Beckman Coulter, CA, USA) and CD279-APC (PD-1) (clone MIH4, BD Pharmingen, CA, USA). Flow cytometric analysis was conducted using a CytoFLEX V5-B5-R3 Instrument (Beckman Coulter, CA, USA) equipped with 405 nm, 488 nm and 638 nm lasers running with CytExpert software v2. Data analysis was performed using FlowJo v10 (LLC Software, SD, USA).

In patients with pulmonary lesions suspicious for lung cancer, transbronchial biopsies or endobronchial ultrasound-guided transbronchial needle aspiration were performed in the same procedure to obtain histological diagnosis in the majority of patients.

### Statistical analysis

Differential cytology and flow cytometry data are described as median with 95% CI. D’Agostino & Pearson test revealed that none of the data were normally distributed. Therefore, Kruskal–Wallis test with Dunn ‘s correction for multiple comparison was applied (GraphPad Prism v8, SD, USA). For correlation of PD1-expression on lymphocytes with PD-L1 expression (TPS [tumor proportion score] and CPS (combined positive score), Spearman correlation was applied. The significance boundary was set to 0.05, no adjustment for multiple testing was performed due to the exploratory character of the study. P-Values were interpreted descriptively.

## Results

Twenty patients with a pulmonary lesion suspicious for lung cancer were enrolled in this prospective trial that was conducted in the pulmonology department, Internal Medicine II, Medical University of Vienna. BAL was performed successfully in all patients.

Histology of 14 patients (male 50%, mean 66 ± 8 years) revealed a squamous cell cancer in 6 patients, an adenocarcinoma in 6 patients, pleomorphic carcinoma in 1 patient and NOS (not otherwise specified) in another patient. Four patients with suspicious peripheral lesion have to been excluded from this trial, as no malignancy was confirmed (n = 2), biopsy revealed a chondrohamartoma (n = 1) or sarcoidosis (n = 1). In two patients, lymphocyte count in the BAL sample was too low for PD1 assessment.

In the patient groups with benign diseases, 10 patients with ILD (male 60%, mean 60 ± 19 years) and 10 patients with asthma (male 30%, mean 53 ± 18 years) were enrolled.

Clinical characteristics are presented in Table 1.

**Table 1:** Clinical characteristics

	Patients with lung cancer n = 14	Patients with ILD n = 10	Patients with asthma n = 10
Sex (male:female)	7:7	6:4	3:7
Age (years; mean ± STD)	66.6 ± 8.6	60.0 ± 14.5	51.5 ± 17.6
Lung function*			
FEV_1_/FVC (%; mean ± SD)	63 ± 14	80 ± 5	71 ± 10
FEV_1_ (%; mean ± SD)	76.6 ± 21.5	84.6 ± 22.1	86.0 ± 25.3
FVC (%; mean ± SD)	93.5 ± 15.9	87.4 ± 21.0	95.9 ± 21.2
*Histology (n; %)*			
Adenocarcinoma	6	–	–
Squamous cell carcinoma	6	–	–
Pleomorphic carcinoma	1	–	–
NOS	1	–	–
*Stage of disease (n; %)*			
IA3	2	–	–
IB	1	–	–
IIB	2	–	–
IIIA	3	–	–
IIIC	1	–	–
IVA	2	–	–
IVB	3	–	–

^*^Not available for 2 patients with lung cancer and 2 patients with asthma

### Results of T lymphocytes isolated by bronchoalveolar lavage

**Fig. 1:**
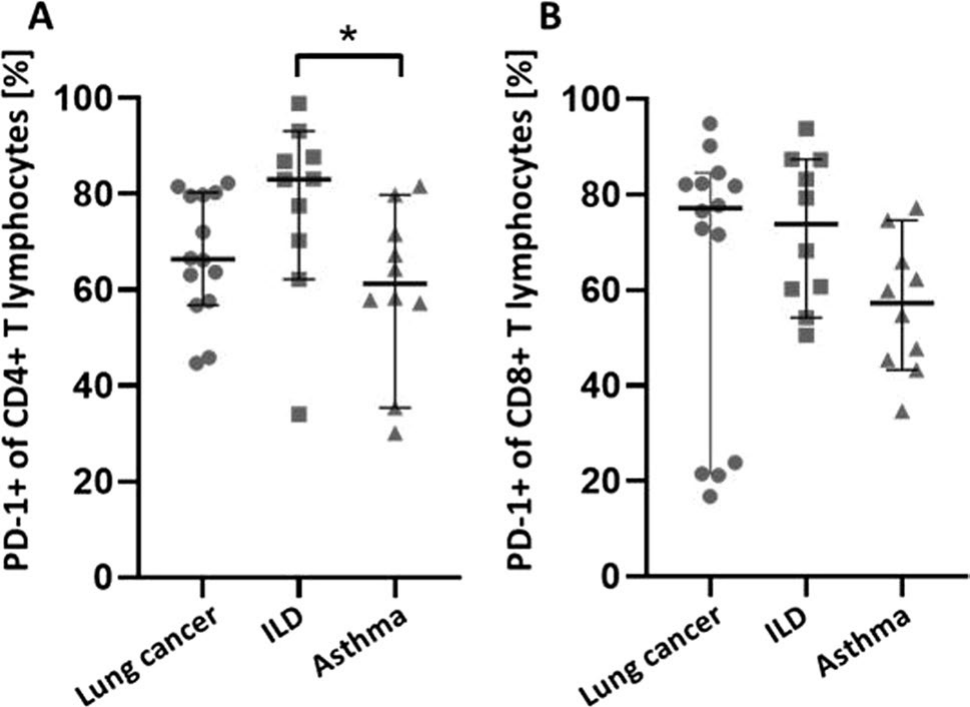
Proportion of **A** PD-1 + CD4 + T lymphocytes and **B** PD-1 + CD8 + T lymphocytes measured in BAL samples obtained from patients with lung cancer (●, n = 14), ILD (■, n = 10) and asthma (**▲**, n = 10). Each data point represents one patient. Data are represented as mean with 95% CI and were tested for statistical differences using Kruskal–Wallis test with Dunn’s multiple comparison. * *p* < 0.05. BAL: bronchoalveolar lavage, ILD: interstitial lung disease, PD-1: programmed cell death protein 1

A significant difference in the proportion of PD-1 + CD4 + cells was found between patients with ILD and asthma (*p* = 0.04) (Table 2, Fig. 1A). The highest median proportion of PD-1 + CD4 + or PD-1 + CD8 + T cells were found in patients with ILD (83.1% [IQR 72.1; 87.5] and 73.8% [IQR 60.3; 86.3]) followed by patients with lung cancer (66.4% [IQR 59; 69] and 77.1% [IQR 35.8; 82.3]) and patients with asthma (61.3% [IQR 57.4; 70.5] and 57.3% [46; 65]) (Table 3, Fig. 1B).

**Table 2:** Differential cytology analysis and flow cytometry in patients with lung cancer (n = 14), patients with ILD (n = 10) and patients with asthma (n = 10). Values are given in median [IQR]

Cell type	Lung cancer	ILD	Asthma
Macrophages (%) [IQR]	87.5 [61.8; 94.8]	82 [55.3; 92.3]	85 [77.3; 91.8]
Lymphocytes (%) [IQR]	8.5 [5; 18]	17.5 [6.5; 43]	15 [8.3; 18]
Neutrophils (%) [IQR]	0 [0; 0.75]	1 [0; 1.75]	0 [0; 1.75]
Eosinophils (%) [IQR]	0 [0; 0]	0 [0; 0]	0 [0; 0.75]
*T lymphocytes subtypes*			
T cells CD3 + (% of all 45 + cells)	4.22 [1.26; 22.2]	7.82 [4.73; 26.95]	14.5 [9.17; 25.38]
T cells CD4 + (% of all 45 + cells)	1.93 [0.355; 8.11]	4.21 [1.92; 14.35]	7.41 [5.91; 18.55]
T cells CD8 + (% of all 45 + cells)	1.49 [0.22; 6.48]	2.6 [1.45; 6.47]	3.35 [1.66; 6]
CD279 + (% of CD4 + cells)	66.4 [59; 69.25]	83.1 [72.1; 87.48]	61.3 [57.38; 70.53]
CD279 + (% of CD8 + cells)	77.1 [35.83; 82.25]	73.75 [60.33; 86.28]	57.25 [45.98; 65]

**Table 3:** In-between group difference in the proportion of PD-1 (CD279) + T cells analysed by flow cytometry

	Mean rank diff	*p* value
*CD279 + (% of CD4 + cells)*		
Lung cancer vs. ILD	− 8.136	0.15
Lung cancer vs. asthma	2.914	> 0.99
ILD vs. asthma	11.05	0.039
*CD279 + (% of CD8 + cells)*		
Lung cancer vs. ILD	− 2.043	> 0.99
Lung cancer vs. asthma	6.657	0.3192
ILD vs. asthma	8.7	0.1523

**Fig. 2:**
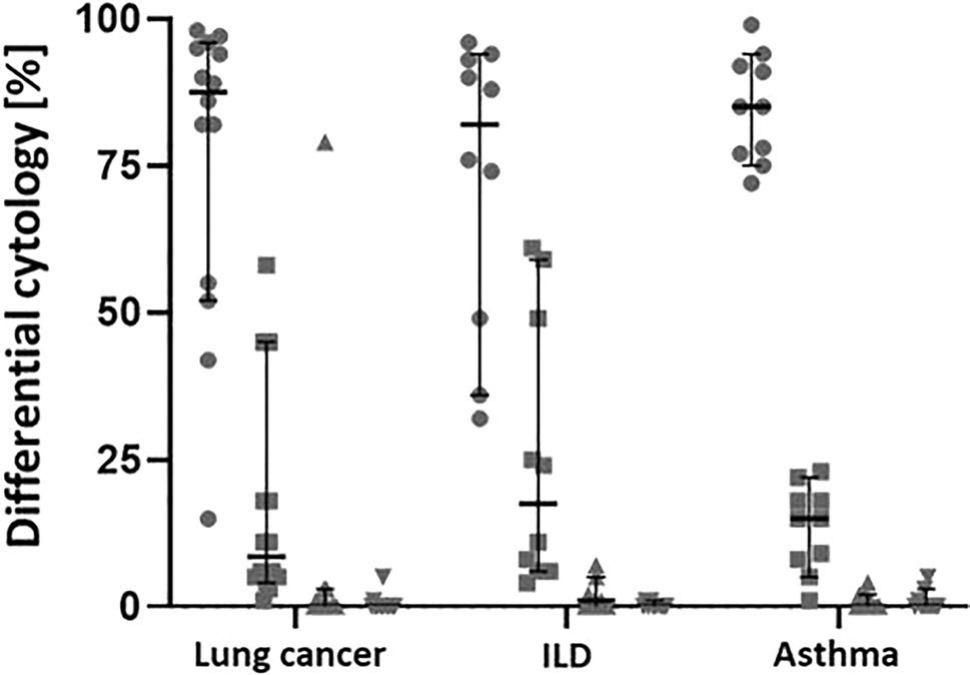
Differential cytology in percent of macrophages (●), lymphocytes (■), neutrophils (**▲**) and eosinophils (**▲**) obtained from BAL samples of patients with lung cancer (n = 14), ILD (n = 10) and asthma (n = 10). Each data point represents one patient. Data are represented as mean with 95% CI and were tested for statistical differences with two-factor ANOVA with Tukey’s multiple comparison procedure. BAL: bronchoalveolar lavage, ILD: interstitial lung disease

There were no significant differences in differential cell frequencies in the BAL samples between the three patient groups (Fig. 2).

### Correlation analysis between PD-1 + T lymphocytes and PD-L1 + tumour cells

There was no statistically significant correlation between PD-1 + CD4 + or PD-1 + CD8 + and TPS or CPS (*p* value > 0.05) (Table 4).

**Table 4:** Spearman correlation of TPS and CPS

Correlation of TPS with	rho	*p* value
CD279 + (% of CD4 + cells)	0.119	0.727
CD279 + (% of CD8 + cells)	0.505	0.113
CPS	0.987	< 0.001

Correlation of CPS with	rho	*p* value
CD279 + (% of CD4 + cells)	0.151	0.699
CD279 + (% of CD8 + cells)	0.561	0.116
TPS	0.987	< 0.001

## Discussion

PD-1 plays an important role in inhibiting immune responses [14]. It is mainly expressed on activated CD4 + T cells, CD8 + T cells, natural killer T cells, B cells, macrophages, dendritic cells (DCs) and monocytes. As it is expressed on the majority of tumor-specific T cells it is responsible for cancer immune escape by binding its ligand PD-L1 on tumor cells. Immune checkpoint inhibitors (ICI, monoclonal antibodies against PD-1 or PD-L1) that target the interaction between PD-1 and PD-L1 has been shown to be a successful anticancer strategy. Nevertheless, the PD-1/PD-L1 blockade is limited by a lack of biomarkers, immune-related toxicity and drug resistance. So far, PD-L1 expression on tumor cells is used as predictor for successful outcome of immunotherapy. However, the association of high PD-L1 expression on tumor cells with treatment response is discussed controversially and may vary among different ICI agents [7]. Moreover, it is questionable, whether the PD-L1 expression that is mainly assessed on biopsy specimens represents the PD-L1 status of the whole tumor as the majority of tumors demonstrate a considerable intra-tumoral heterogeneity in PD-L1 expression [15, 16]. As PD-1 presents the counterpart of PD-L1, it can be hypothesized, that besides the PD-L1 expression on tumor cells, the proportion of PD-1+ lymphocytes in lung cancer patients may have impact on immunotherapy action and may present a predictor for immunotherapy response.

Thus, it is crucial to elucidate the expression of PD-1 on lymphocytes in the microenvironment of lung cancer. So far, there are only limited data related to the PD-1expression on T lymphocytes in the bronchoalveolar lavages of patients with lung cancer. Kwiecien et al. has already demonstrated that the proportion of CD8 + cells with the expression of PD-1 and CTLA-4 were elevated in the bronchoalveolar lavage fluid of the lung affected by lung cancer compared to the healthy lung or compared to the blood [10].

This current trial describes for the first time the PD-1 expression on T lymphocytes in the BAL of lung cancer patients compared to patients with benign diseases. The highest median proportion of PD1 + CD4 + or PD1 + CD8 + cells were found in patients with ILD. The overexpression of PD-1 on T lymphocytes can be explained by the fact that PD-L1 is not only expressed by tumor cells but is aberrantly expressed on human fibroblasts of lung tissue samples [13, 19]. Besides an overexpression of PD-L1, an upregulated expression of PD-1 in CD4 + T cells in blood samples and an increased PD-1 expression in idiopathic lung fibrosis (IPF) human lung biopsies were described [17, 18]. It is hypothesized that PD-L1/PD-1 interaction mediates fibroblasts to myofibroblast transition and potentially the development of pulmonary fibrosis [20]. Therefore, trials evaluating the potential therapeutic effect of PD-1/PD-L1 blockades on IPF are of great interest.

The median proportion of PD-1 + CD4 + or PD-1 + CD8 + cells in patients with lung cancer was found to be 66% and 77%, respectively. This result was similar to the finding of Kwiecien et al. that found a median proportion of PD-1 + CD4 + of 52% and of PD-1 + CD8 + cells of 68% [10]. In our study, there was no statistically significant difference in the proportion of PD-1 + T lymphocytes between patients with lung cancer and benign lung diseases. Moreover, we did not find a statistically significant correlation between PD-1 + CD4 + or PD-1 + CD8 + and TPS or CPS. Nevertheless, further trials are needed that evaluate the impact and predictive character of PD-1 + T lymphocytes in BAL of lung cancer patients for immunotherapy response in addition to analysing tumor samples for PD-L1 expression. In addition, the percentage of PD-1 + T lymphocytes in BAL should be evaluated with regard to the tumor size, tumor location and histology. For example, Kwiecien I et al. found a higher proportion of PD-L1 lymphocytes in BAL of patients with squamous cell carcinoma when compared to adenocarcinoma patients [10].

The lowest median proportion of PD1 + CD4 + or PD1 + CD8 + cells was found in patients with asthma. It is assumed, that the PD-1/PD-L1 axis also plays a role in the chronic inflammatory processes in asthma. Whereas PD-L1 seems to have a pro-asthmatic role, PD-1 and PD-L2 may have a protective role and their agonists may be used to ameliorate the airway hypersensitivity [21, 22]. However, the clinical relevance of the PD-1/PD-L1 pathway and its potential therapeutic role in asthma has to be investigated in further trials.

One limitation of this prospective trial is the small number of participants. The greater the sample size, the more likely we are to find a statistically significant difference between groups. Although this trial did not reveal a statistically significant difference in BAL samples between patients with malignant and benign lung diseases, there was evidence of a higher number of PD-1 + T lymphocytes in "proliferating" diseases, such as lung carcinoma or interstitial diseases. Larger trials are needed to elucidate the impact of PD-1 + T lymphocytes in these diseases.

In summary, the proportion of PD-1 + T cells did not differ in BAL samples of patients with lung cancer in comparison to patients with ILD or asthma. The highest median proportion of PD-1 + T lymphocytes were found in ILD patients followed by lung cancer patients. IPF and lung cancer share many pathogenic similarities [23], assuming a common PD-1/PD-L1 mechanism. However, the PD-1 pathway is more likely utilized by cancer cells to escape the surveillance of the immune system, whereas in lung fibroblasts, PD-1 seems to be more a profibrotic factor. As the number of patients recruited in each arm was too low to draw firm conclusions, further trials are warranted.

## Data Availability

The datasets generated during and/or analysed during the current study are available from the corresponding author on reasonable request.
